# Statement on the update of WHO’s working definitions and tracking system for SARS-CoV-2 variants of concern and variants of interest

**Published:** 2023-04

**16 March 2023 -** WHO has updated its tracking system and working definitions for variants of SARS-CoV-2, the virus that causes COVID-19, to better correspond to the current global variant landscape, to independently evaluate Omicron sublineages in circulation, and classify new variants more clearly when required.

SARS-CoV-2 continues to evolve. Since the beginning of the COVID-19 pandemic, multiple variants of concern (VOCs) and variants of interest (VOIs) have been designated by WHO based on their assessed potential for expansion and replacement of prior variants, for causing new waves with increased circulation, and for the need for adjustments to public health actions.

Based on comparisons of antigenic cross reactivity using animal sera, replication studies in experimental models of the human respiratory tract, and evidence from clinical and epidemiological studies in humans, there is consensus among experts in WHO’s Technical Advisory Group on SARS-CoV-2 Virus Evolution (TAG-VE) that compared to previous variants, Omicron represents the most divergent VOC seen to date. Since its emergence, Omicron viruses have continued to evolve genetically and antigenically with an expanding range of sublineages, which so far have all been characterized by properties of evasion of existing population immunity and a preference to infect the upper respiratory tract (versus lower respiratory tract), as compared to pre-Omicron VOCs.

The Omicron viruses account for over 98% of the publicly available sequences since February 2022 and constitute the genetic background from which new SARS-CoV-2 variants will likely emerge, although the emergence of variants derived from previously circulating VOCs or of completely new variants remains possible. The previous system classified all Omicron sublineages as part of the Omicron VOC and thus did not have the granularity needed to compare new descendent lineages with altered phenotypes to the Omicron parent lineages (BA.1, BA.2, BA.4/BA.5). Therefore, from 15 March 2023, the WHO variant tracking system will consider the classification of Omicron sublineages independently as variants under monitoring (VUMs), VOIs, or VOCs.

WHO is also updating the working definitions for VOCs and VOIs. The main update consists in making the VOC definition more specific, to include major SARS-CoV-2 evolutionary steps that require major public health interventions. For the updated definitions, please visit the WHO variant tracking website.

In addition, going forward, WHO will assign Greek labels for VOCs, and will no longer do so for VOIs.

With these changes factored in, Alpha, Beta, Gamma, Delta as well as the Omicron parent lineage (B.1.1.529) are considered previously circulating VOCs. WHO has now classified XBB.1.5 as a VOI.

WHO will also continue to issue regular risk assessments for both VOIs and VOCs (see latest risk assessment for XBB.1.5).

WHO emphasizes that these changes do not imply that the circulation of Omicron viruses no longer pose a threat to public health. Rather, the changes have been made in order to better identify additional or new threats over and above those posed by the current Omicron viruses in circulation.

Available from: https://www.who.int/news/item/16-03-2023-statement-on-the-update-of-who-s-working-definitions-and-tracking-system-for-sars-cov-2-variants-of-concern-and-variants-of-interest

